# HIV-1 Viral Loads Are Not Elevated in Individuals Co-infected With *Schistosoma* spp. After Adjustment for Duration of HIV-1 Infection

**DOI:** 10.3389/fimmu.2018.02005

**Published:** 2018-09-06

**Authors:** Soledad Colombe, Paul L. A. M. Corstjens, Claudia J. de Dood, Donald Miyaye, Ruth G. Magawa, Julius Mngara, Samuel E. Kalluvya, Lisette van Lieshout, Govert J. van Dam, Jennifer A. Downs

**Affiliations:** ^1^Department of Medicine, Center for Global Health, Weill Cornell Medicine, New York, NY, United States; ^2^Department of Cell and Chemical Biology, Leiden University Medical Center, Leiden, Netherlands; ^3^National Institute for Medical Research, Mwanza, Tanzania; ^4^Department of Medicine, Bugando Medical Centre, Mwanza, Tanzania; ^5^Department of Parasitology, Leiden University Medical Center, Leiden, Netherlands

**Keywords:** *Schistosoma* spp., HIV-1, Viral load, Plasma HIV-1 RNA, Tanzania

## Abstract

Studies of the role of *Schistosoma* co-infections on plasma HIV-1 RNA (HIV-1 viral load) have yielded incongruent results. The role of duration of HIV-1 infection on the link between *Schistosoma* and HIV-1 viral load has not been previously investigated. We aimed to assess the impact of HIV-1/*Schistosoma* co-infections on viral load in Antiretroviral Treatment (ART)-naïve HIV-1 infected people taking into account the duration of HIV-1 infection. We describe 79 HIV-infected outpatients greater than 18 years of age who had never used ART in Mwanza, Tanzania. Schistosomiasis testing was done by urine and stool microscopy and by serum *Schistosoma* circulating anodic antigen (CAA) testing. *Schistosoma* positivity was defined as having either test positive. We conducted univariable and multivariable linear regressions to assess the relationship between *Schistosoma* infection and the log_10_ of viral load. Duration of HIV infection was calculated using the first measured CD4^+^ T-cell (CD4) count as a function of normal CD4 count decay per calendar year in drug naïve individuals. An active *Schistosoma* infection was demonstrated in 46.8% of the patients. The median log_10_ viral load was 4.5[3.4–4.9] log_10_ copies/mL in *Schistosoma* uninfected patients and 4.3[3.7–4.6] log_10_ copies/mL in *Schistosoma* infected patients. *Schistosoma* co-infection was negatively associated with the log_10_ of viral load after adjustment for *Schistosoma* intensity as measured by CAA, CD4 counts at time of testing, and duration of HIV-1 infection (β = −0.7[−1.3;−0.1], *p* = 0.022). *Schistosoma* co-infection was not associated with viral load in univariable analysis. There was also no interaction between *Schistosoma* positivity and duration of HIV-1 infection. Our study is the first, to our knowledge, to report adjustment for duration of HIV-1 infection when analyzing the relationship between HIV-1 viral load and *Schistosoma* spp. We found that time infected with HIV-1 has a major effect on the relationship between HIV-1 viral load and *Schistosoma* infection and may be a critical explanatory factor in the disparate findings of studies on HIV-1 viral load and schistosomiasis. The log_10_ viral load difference found indicates that *Schistosoma* co-infection does not make HIV progression worse, and could possibly lead to slower HIV disease progression.

## Introduction

Although Africa makes up just 15% of the worldwide population, it is burdened by 70% of the world's 36.7 million HIV infections and 91% of the world's 240 million *Schistosoma* infections (1, 2). The 2013 Global Burden of Disease Study estimated that schistosomiasis alone causes 2.6 million disability-adjusted life years (DALYs) lost annually, while HIV infection alone causes 66.7 million DALYs (3). Of note, DALY calculations for HIV and schistosomiasis account for each infection separately and do not consider impacts that they may have on one another.

A growing body of animal and human studies supports a complex relationship between HIV and schistosomiasis. Animal studies suggest that schistosomiasis may alter immune control of viral co-infections, facilitating viral reactivation and replication (4–6). However the role of *Schistosoma* spp. co-infections on plasma HIV-1 RNA (HIV-1 viral load) is still unclear, with various studies reporting higher, lower, or equivalent HIV-1 viral loads in those with *Schistosoma* co-infection (7–15). Within this body of evidence, the longest time that people with HIV and *Schistosoma* co-infection have been followed was approximately 24 months. Our group has recently documented an unexpected improved long-term HIV disease-free survival in those with HIV/*Schistosoma* co-infections at time of HIV-1 seroconversion (16). This suggests that chronic *Schistosoma* infection may downregulate HIV-1 viral replication even though the opposite has been observed during acute infection (6, 15), or that time infected with HIV may have been a confounder in studies that examine the link between *Schistosoma* spp. and HIV-1 viral load.

We thus aimed to assess the impact of HIV-1/*Schistosoma* spp. co-infections on viral load in Antiretroviral Treatment (ART)-naïve HIV-1 infected people taking into account the duration of HIV-1 infection. To investigate this question, we designed a study situated within an outpatient HIV clinic at which approximately 30% of individuals are *Schistosoma* -infected (17, 18), and enrolled patients who would shortly be starting ART.

## Methods

### Study participants and enrollment

This study was conducted in April and May 2015 in an HIV outpatient clinic at Bugando Medical Centre (BMC) in Mwanza. The participants were HIV-infected adults greater than 18 years of age who had never used ART according to clinic records and patient report.

Eligible patients provided a single urine and stool sample for schistosomiasis testing by microscopy in order to determine which species of schistosomes were present, as well as serum for quantitation of *Schistosoma* Circulating Anodic Antigen (CAA), which measures the intensity of infection (19). Plasma was also collected for viral load measurement. Additional information was extracted from the HIV clinic database and the patient's chart.

### Laboratory methods

Microscopic testing was performed on 10 mL of urine by the filtration technique and on feces following the Kato Katz method. Testing was performed by parasitologists at the National Institute of Medical Research (NIMR) in Mwanza, Tanzania. Five Kato Katz slides using 41.7 mg of stool per slide were used, which has been shown to have a sensitivity comparable to collecting three stool samples on different days (20). CAA testing was performed at NIMR in Mwanza as previously described, using a positivity threshold of 30 pg/mL (dry reagent SCAA20 assay format) (19). In order to maximize sensitivity of testing for *Schistosoma* infections in this HIV-infected population, we used a composite score in which we defined *Schistosoma* infection as having either a microscopy or CAA positive test. Plasma viral load was quantified using the COBAS® AmpliPrep/COBAS® TaqMan® HIV-1 Test (Roche Molecular Systems Inc., Pleasanton, California, USA) at the BMC clinical laboratory, with a lower limit of detection of 20 copies/mL.

### Statistical analysis

Data was double entered, verified and cleaned using Microsoft Excel 2013 and analysis was performed using STATA version 13. Categorical data were described with proportions and continuous data were described with median and interquartile range. Chi-square tests and *t*-tests were used to compare presence of demographic and clinical factors between those co-infected with *Schistosoma*/HIV-1 and those infected with HIV-1 only. Both viral loads and CAA values had extreme outliers and were skewed to the right. We thus used the log_10_ of viral load and natural log of CAA, by convention. Univariable and multivariable linear regressions were used to assess the relationship between *Schistosoma* infection and the log_10_ of viral load. We also assessed the association between *Schistosoma* infection and CD4 counts. All variables significantly associated with the outcome in the univariable analysis were included in the multivariable analysis. A stepwise analysis was conducted for the multivariable analysis. A quantile regression was used to assess the interaction between *Schistosoma* infection and duration of HIV-1 infection and its impact on the difference in median of the log_10_ of viral load.

Time infected with HIV was defined using the first CD4^+^ T-cell counts (CD4 counts) reported at the clinic. This method has been previously used with some variation (21–25). Due to similarity in the available data and study setting, we used the method of Forbi et al. (23). The CD4 counts at time of enrollment at the clinic were used to approximate the time delay between HIV infection and enrollment as a function of normal CD4 decay per calendar year in drug naïve individuals. The normal reference values of CD4 counts in healthy Tanzanians have been estimated at a median of 596.5 [291.2–1278.9] cells/μL for men and 764.5 [288.5–1406.8] cells/μL for women (26). In addition, the most prevalent HIV infecting clade in the Lake Zone in Tanzania is clade A (27) and in Mwanza, Tanzania, Clade A and D viruses make up the majority (34 and 28% respectively) of HIV infections (28). This is similar to proportions found on the Ugandan side of the lake, in Rakai (29) and Entebbe (30) and the overall normal CD4 decay per calendar year, across all clades, has been shown to be approximately 34.5 cells/μL per year in Rakai (29).

Starting from the upper range of the normal reference values for CD4 counts, we modeled decay by the square-root function as suggested by Kiwanuka et al. (29), which meant we subtracted 5.87 cells^1/2^/μL^1/2^ from 35.76 cells^1/2^/μL^1/2^ for men and 37.51 cells^1/2^/μL^1/2^ for women per calendar year period until the square root of the first CD4 count reported at the clinic was reached. The time period for this to happen was considered to be the estimated period between HIV-1 acquisition and enrollment at the clinic. The time between the first CD4 count reported at the clinic and the date of viral load testing was then added to this variable to obtain the duration of HIV-1 infection. This led to an estimated median time from seroconversion to enrollment of 2.5[1.7–3.0] years, which is similar to the median time from seroconversion to enrollment estimated by our group within the Kisesa Lake region cohort using mid-dates between two serosurveys as date of seroconversion (manuscript submitted). Finally, to look at the interaction between *Schistosoma* infection and time infected with HIV, we categorized the latter using tertiles.

### Ethical considerations

All participants were recruited after providing written informed consent in accordance with the declaration of Helsinki. Clearance was obtained from the joint CUHAS/BMC Research Ethics Committee, the National Institute for Medical Research in Dar es Salaam, Tanzania, and Weill Cornell Medical College, New York. All clinical data were made available immediately to clinicians and recorded in the patient's medical record. All patients with *Schistosoma* infection received praziquantel 40 mg/kg free of charge.

## Results

We enrolled 83 HIV-infected patients who presented at the clinic and had never initiated ART. Thirty-seven out of seventy-nine (46.8%) were positive for *Schistosoma* spp. either by CAA or microscopy test. 33/81 (40.7%) were positive by CAA and the median CAA was 18.3[5.6–517.2] pg/mL. The distribution of CAA values was skewed to the right. Therefore we log-transformed it and the median ln CAA was 3.0[1.9–6.3] ln pg/mL. None had a positive urine microcopy and among those with positive stool microscopy (20/81–24.7%), the median of the mean egg count was 21.6[4.8–52.8] eggs/gram. Seventeen patients were CAA positive but urine and stool negative, while 4 patients were stool positive but CAA negative. Patients had a median age of 36[29-41] years, and 67/83 (80.7%) were female. Median CD4 counts at enrollment was 504[395-749] cells/μL and median CD4 counts at time of viral load testing was 455[328-614] cells/μL.

Patients had enrolled in the HIV clinic a median of 2.5[1.7–3.0] years after acquiring HIV, and provided viral loads for this study a median of 3.7[3.0–5.7] (minimum = 1.7, maximum = 12.0) years after acquiring HIV. The median viral load was 21,670.5[2,852.0–56,160.0] copies/mL, or 4.3[3.5–4.7] log_10_ copies/mL. The median log_10_ viral load was 4.5[3.4–4.9] log_10_ copies/mL in *Schistosoma* uninfected patients and 4.3[3.7–4.6] log_10_ copies/mL in *Schistosoma* infected patients. The main variables are presented in Table 1 by *Schistosoma* infection status.

**Table 1:** Characteristics of the 79 HIV-1 infected patients tested for *Schistosoma* infection who had never initiated ART.

	***Schistosoma* free (*n* = 42) n/N(%) or median[IQR]**	***Schistosoma* infected (*n* = 37) n/N(%) or median[IQR]**	***p*-value**
Female sex	37/42 (88.1%)	27/37 (73.0%)	0.087
CD4 count at time of VL testing (cells/μL)	475 [314.5–658.5]	439 [336-566]	0.66
Log_10_ of viral load (copies/mL)	4.5 [3.4–4.9]	4.3 [3.7–4.6]	0.21
Age in years	36 [28-42]	35 [30-40]	0.81
Years infected with HIV-1 (as a continuous variable)^*^	3.7 [3.0–6.5]	3.7 [3.0–5.3]	0.72
Years infected with HIV-1^*^			0.82
<3 years	12/40 (31.6%)	12/36 (30.3%)	
3–5 years	13/40 (28.9%)	13/36 (39.4%)	
>5 years	15/40 (39.5%)	11/36 (30.3%)	

* *3 patients did not have any CD4 count reported, precluding calculation of the years infected with HIV*.

After univariable linear regression, female sex, higher CD4 counts and longer time infected with HIV-1, were all significantly associated with lower log_10_ of the viral load. *Schistosoma* positivity and ln of CAA were not associated with log_10_ of the viral load (Table 2).

**Table 2:** Results of the univariable analysis with log_10_ of viral load as a continuous outcome.

	**Slope coefficient (95%CI)**	***p*-value**
Sex (Female)	−0.6 [−1.1; −0.08]	0.024
CD4 count at time of VL testing		
<200 cells/μL	Ref	
200–500 cells/μL	−0.7 [−1.3; −0.02]	0.042
500–1,000 cells/μL	−1.2 [−1.9; −0.5]	0.001
>1,000 cells/μL	−1.06 [−2.0; −0.1]	0.024
Schistosoma positivity	−0.15 [−0.6;0.3]	0.49
Age (in years)	0.02 [−0.005; 0.04]	0.13
Ln of CAA in ln pg/mL	0.006 [−0.07;0.08]	0.86
Years infected with HIV-1	−0.1 [−0.2; −0.03]	0.008

**Figure 1:**
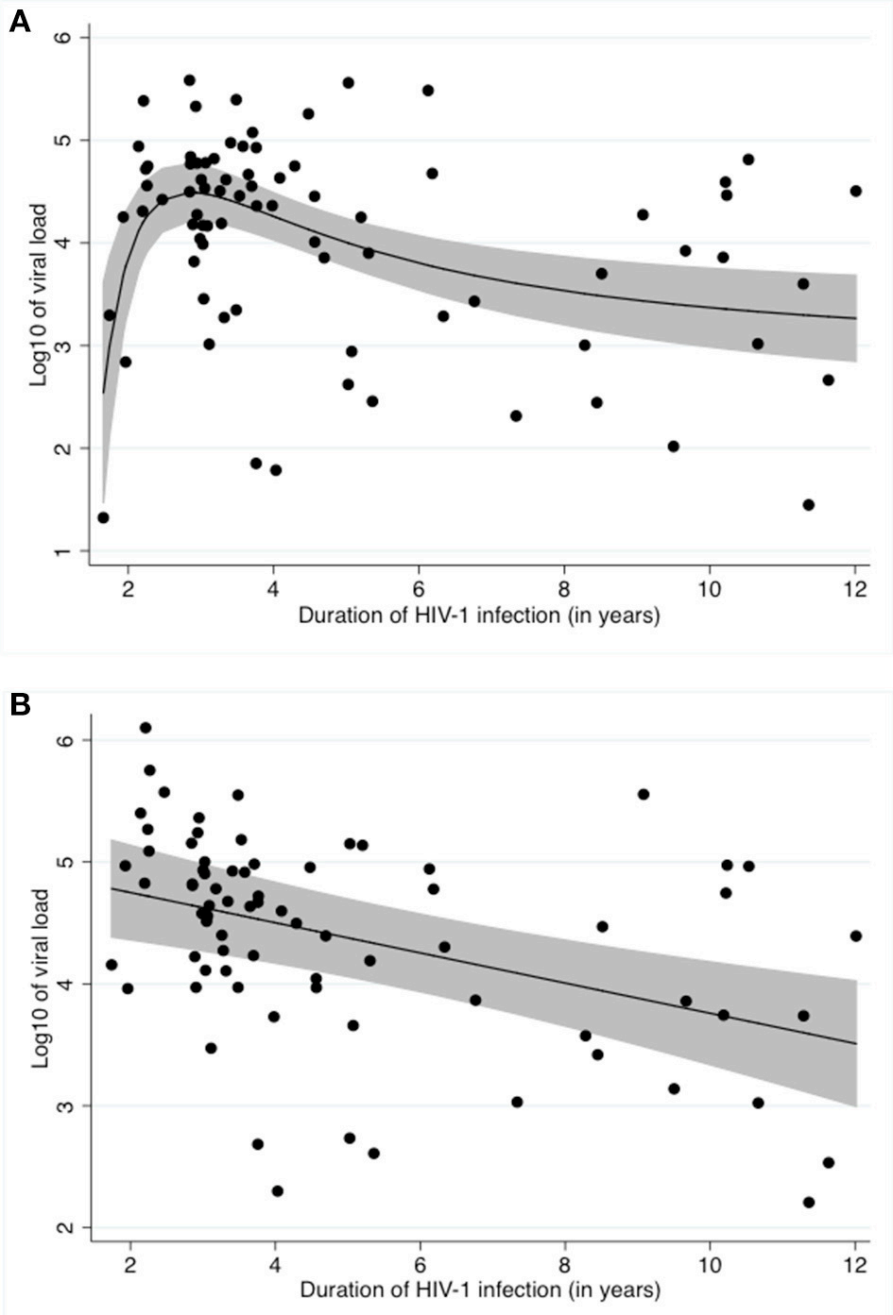
Relationship between log_10_ of the viral load and time infected with HIV **(A)** Unadjusted, **(B)** Adjusted for *Schistosoma* status, ln of CAA and CD4 counts. **(A)** shows the crude relationship between log_10_ of viral load and duration of HIV-1 infection. A fractional polynomial was fitted to the data. The black line represents the predicted log_10_ of viral load after applying the resulting function to the data. The grey area represents the 95% confidence limits around the fitted values. The black dots represent the residuals. **(B)** shows the relationship between log_10_ of viral load and duration of HIV-1 infection after adjustment for *Schistosoma* infection status, ln of CAA and CD4 counts using a fractional polynomial. The black line represents the predicted log_10_ of viral load after applying the resulting function to the data. The grey area represents the 95% confidence limits around the fitted values. The black dots represent the residuals.

Our unadjusted data shows a typical relationship between viral load and time infected with HIV-1, as described by other studies (Figure 1A) (31).

After stepwise multivariable analysis, the best-fit model for log_10_ viral load included *Schistosoma* positivity, ln of CAA, CD4 counts at time of study enrollment and time infected with HIV-1. *Schistosoma* positivity was negatively associated with the log_10_ of viral load after adjustment (−0.7[−1.3;−0.1], *p* = 0.022). Sex and age were not part of the best-fit model. The best-fit model is presented in Table 3. Of note, this model includes both the *Schistosoma* infection status as a binary variable and also the natural log of the CAA value to assess whether the intensity of the *Schistosoma* infection impacted the viral load. The relationship between duration of HIV-1 infection and log_10_ of viral load adjusted for *Schistosoma* status, ln of CAA, and CD4 counts is shown in Figure 1B.

**Table 3:** Results of the multivariable linear regression with log_10_ of viral load as a continuous outcome.

	**Slope coefficient (95%CI)**	***p*-value**
*Schistosoma*-infection status	−0.7 [−1.3; −0.1]	0.022
Ln of CAA in ln pg/mL	0.08 [−0.007;0.2]	0.070
CD4 counts at VL testing		
<200 cells/μL	Ref	
200–500 cells/μL	−0.7 [−1.2; −0.1]	0.022
500–1,000 cells/μL	−1.2 [−1.8; −0.6]	<0.001
>1,000 cells/μL	−1.7 [−2.7; −0.7]	0.001
Years infected with HIV-1	−0.1 [−0.2; −0.06]	<0.001

**Figure 2:**
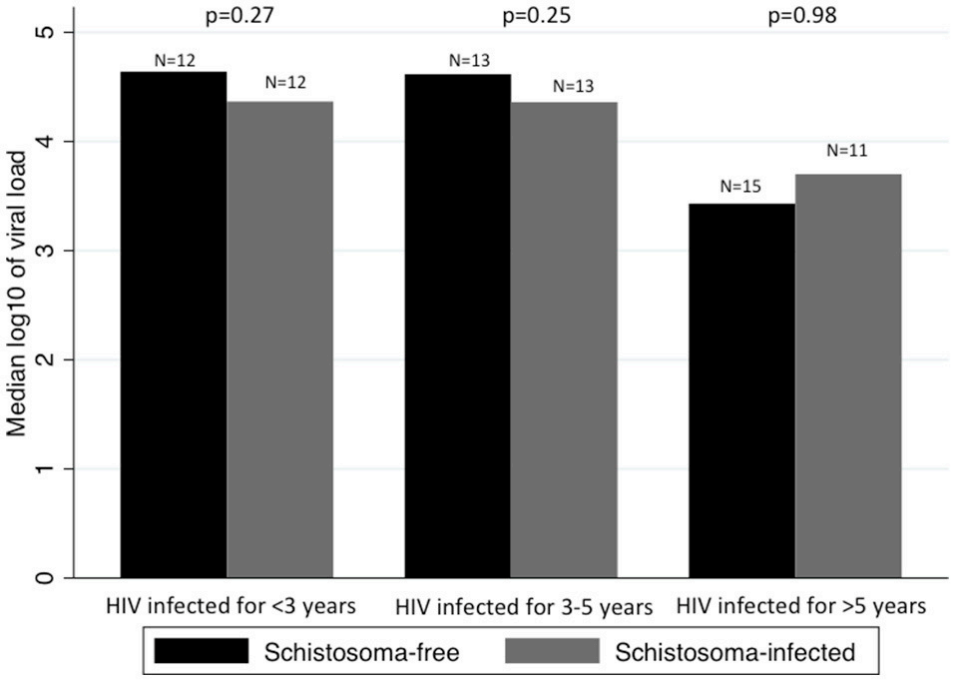
Comparison between median viral loads by time infected with HIV-1 and by *Schistosoma*-infection status. Panel shows the median log_10_ of viral load in *Schistosoma*-free and *Schistosoma*-infected patients, by category of duration of HIV-1 infection. The difference in median log_10_ of viral load was assessed by rank-sum test. There was no difference in median log_10_ of viral load between *Schistosoma*-free and *Schistosoma*-infected, regardless of the duration of HIV-1 infection.

When looking at whether the difference in median log_10_ of viral load between *Schistosoma* infected and uninfected patients changed over time, we found no statistical significance. Figure 2 assesses the difference in median of log_10_ viral load between *Schistosoma* infected and uninfected patients within each HIV-1 infected time category.

We assessed the impact of time on the difference in median of log_10_ viral load between *Schistosoma* infected and uninfected patients using a quantile regression with an interaction term between *Schistosoma* status and duration of HIV-1 infection. In other words, we assessed whether the difference in viral loads between *Schistosoma* infected and uninfected patients seen in people HIV infected for 3–5 years is significantly different from the difference seen in people HIV infected for <3 years, and again for >5 vs. <3 years. The quantile regression showed no significant interactions between *Schistosoma* status and time infected with HIV-1.

In our cohort, *Schistosoma* status was not associated with CD4 counts (slope coefficient = −48.2 [−184.8; 88.4], *p* = 0.48).

## Discussion

Our study showed that current infection with *Schistosoma* spp. was associated with significantly lower HIV-1 viral loads after adjusting for CD4 counts and time infected with HIV-1. The viral load difference of 0.7 log_10_ copies/mL would be expected to lead to over a 60% decrease in risk of HIV transmission and of reaching AIDS-related death for *Schistosoma* -co-infected patients compared to *Schistosoma* -free patients (32). This is in close alignment with the 82% decrease in risk of reaching lower CD4 counts and/or death found by our group (16) using a different analysis technique and in a different population. Taken together, these findings suggest the possibility that long-term HIV outcomes may be positively affected by *Schistosoma* infection.

Our study is the first, to our knowledge, to report adjusting for duration of HIV-1 infection when studying the relationship between HIV-1 viral load and *Schistosoma* spp. Time infected with HIV-1 was a main confounder of the relationship between HIV-1 viral load and *Schistosoma* infection. It is well known that CD4 counts and viral load in HIV-1 infected individuals, and the rates at which they change, differ over time (33–35), which makes it difficult to compare changes in these parameters between two individuals at different periods of their HIV-1 infection (36). Duration of HIV-1 infection may thus be a critical explanatory factor in the disparate findings of studies on HIV-1 viral load and *Schistosoma* infections (7–15), as suggested by Walson et al. (36). Other studies' lack of control for duration of HIV-1 infection may have hindered accurate analysis of the relationship between HIV-1 and *Schistosoma* infections. This may also be true for most studies looking at the relationship between other helminths and HIV-1 infections.

In addition, the control for ART initiation has been inconsistent in most studies. Many investigators have either assumed that all participants were ART naïve due to past limited availability of ART, or have not mentioned ART intake at all in their studies (7, 8, 10, 11, 15). Importantly, given the drastic effect of ART on CD4 counts and HIV-1 viral load (31), failure to account for ART intake in even a few individuals could have biased the results. By choosing patients who had been followed in the HIV outpatient clinic and would be beginning ART in the near future, and by measuring their viral load before initiation of ART, we avoided having the relationship between *Schistosoma* spp. and HIV-1 viral load distorted by the effect of ART on viral loads.

The association between sex and viral load on univariable analysis is unsurprising. Male sex has previously been shown to be associated with higher viral loads (37–39). The decrease in viral load with increasing CD4 counts has also been previously documented in sub-Saharan Africa (40, 41). The overall decrease in viral load over time infected with HIV-1 is logical, as too few of our patients have been infected with HIV-1 for over 10 years to see the late-stage re-increase in viral loads.

Our results are to be interpreted in light of some limitations. The sample size was small due to the expense and relative unavailability of viral load testing at a time when viral load testing was just becoming available at our clinic. In addition, despite not being significant in our study or in a study in South Africa (42), others have shown an impact of *Schistosoma* spp on CD4 counts (8, 10, 11, 16, 43–45), potentially biasing our calculation of the duration of HIV infection. Assessing this relationship using other methods to determine the length of HIV infection would be useful. Nonetheless, the facts that significance is still attained and that variables expected to impact viral load do impact it strengthen confidence in the quality and accuracy of our analysis. Larger, longitudinal studies would be useful in order to investigate the potential interactions between duration of HIV-1 infection and *Schistosoma* infection and its effect on HIV-1 viral load. Investigating the effect of praziquantel treatment would also be of interest given studies that suggest that tissue lesions may not regress after treatment (46).

In conclusion, our work demonstrates that individuals with HIV-1 and *Schistosoma* co-infections had lower viral loads than those with HIV-1 alone, when accounting for time infected with HIV-1. The difference in viral load suggests that *Schistosoma* infection may not lead to worse HIV-1 outcomes nor higher HIV-1 transmission. Future studies of interactions between HIV-1 and *Schistosoma* spp. should account for the duration of HIV-1 infection in their analyses.

## Data availability

The raw data supporting the conclusions of this manuscript will be made available by the authors, without undue reservation, to any qualified researcher.

## Author contributions

SC participated in data collection, analyzed the data and wrote the original manuscript. CdD, PC, and GvD assisted with laboratory and data analysis and reviewed the final manuscript. DM, RM, and JM conducted laboratory testing and review the final manuscript. LvL assisted with data analysis and reviewed the final manuscript. SK designed the study and reviewed the final manuscript. JD designed the study, enrolled the patients, analyzed the data and wrote the original manuscript. All authors have reviewed and approved the current submission.

### Conflict of interest statement

The authors declare that the research was conducted in the absence of any commercial or financial relationships that could be construed as a potential conflict of interest.
